# COVID-19: better trustworthiness of clinical evidence through clinical trials and systematic reviews

**DOI:** 10.1590/1516-3180.2019.138311062020

**Published:** 2020-07-17

**Authors:** Álvaro Nagib Atallah

**Affiliations:** I MD, PhD. Full Professor and Head of the Discipline of Emergency Medicine and Evidence-Based Medicine, Universidade Federal de São Paulo (UNIFESP), and Director of Cochrane Brazil, São Paulo (SP), Brazil.

Recently, two major medical journals, The Lancet and New England Journal of Medicine, each withdrew an article on coronavirus from their databases^1^ and the World Health Organization (WHO) cancelled and restarted a clinical trial on the use of hydroxychloroquine for treating the COVID-19 disease. Soon afterwards, the WHO stated that the possibility of transmission of severe acute respiratory syndrome coronavirus 2 (SARS-CoV-2) by asymptomatic individuals would only be rare, but then quickly denied what had been declared.

Announcements of cures for COVID-19 are appearing frequently. However, there is a clear simultaneous association between such news and the share values of drug-manufacturing laboratories that are promising miraculous cures on the basis of just a few cases that were poorly or barely documented.

It seems that the virus is also affecting the health sciences, the reputations of renowned journals, the state of political debate and the institutions responsible for preserving the population’s state of physical, mental and social wellbeing, i.e. people’s health.

All of this is easy to understand given the gravity of the tragic context. Moreover, it has occurred because clinical research not only requires clinical competence but also needs great detachment, time, impartiality and good evidence (proof) coming from other careful research.

Healthcare practices and evidence-based medicine require the use of reasoning. However, we need to be prepared not only to use rational thinking but also to avoid situations in which interests, emotions, fantasies, charlatanism and so on might get in the way of rationality and free scientific thought. Through this, professional practice can be based on good evidence, i.e. valid scientific proof, that interventions will have a higher chance of providing more benefit than harm.

For more than 30 years, together with many colleagues, we have striven to improve research, teaching and application of evidence-based medicine in Brazil and internationally. The results have been satisfying, because teaching and research within evidence-based medicine has had clear scientific, didactic, economic and juridical impacts, both in Brazil and around the world, over these last decades.

The thirst and respect for scientific evidence seen among many journalists in Brazil during this pandemic has been remarkable. It shows that our continuous collective work over this 35-year period has been worthwhile.

The charlatans who always appear at times of despair have had short lives with their vested-interest fake news, thanks to the now-established cultural foundation of demand for valid scientific evidence, coming from many different sectors of society.

Judicialization of healthcare in Brazil, in which judges have been asked to make rulings in cases based on emotional blackmail, for expensive treatments without proof of effectiveness to be released, under the justification that “without this, the patient will die”, has largely been rationalized, with major savings of public expenditure. This has occurred through requirements for good evidence of effectiveness, efficiency and safety to be presented before permission for these therapies to be released through judicial petitions is granted. In this manner, the groundbreaking concept of evidence-based rights to healthcare has been created in Brazil. In this regard, we have trained around 3,000 judges, prosecutors and attorneys through distance-learning courses sponsored by the Brazilian Ministry of Health.

Unfortunately, for a variety of understandable reasons, the great pioneering experience of doctors in Wuhan, China, regarding COVID-19 could not be properly followed up with enough adequate clinical research methodologies, to generate valid proof, given the lack of time.

There was no time for good orchestration of scientific production that would properly take into account the basic principles for obtaining and evaluating valid evidence. It also has to be recognized that entire impact of this pandemic has been felt within a period of only six months. Most of the data have been retrospective.

A good clinical trial, with more than 1000 cases, would require several years of intense dedicated work. Cochrane systematic reviews take two to four years to be published. The Cochrane Collaboration is now promoting rapid reviews of lower sophistication on matters of urgent interest, within the fight against COVID-19.

If a new disease emerges, there is a need to know the following, for example:

- How to diagnose the disease
- What the clinical condition and its diagnostic tests are, and what usefulness and credibility the results from these tests have
- What the risk factors for individuals to become infected are
- What the risk factors for occurrences of severe outcomes are: for example, shock and kidney failure
- What the risk factors signaling severe forms of the disease and death are
- How to minimize complications of the disease, such as thromboembolism, cytokine storms, inflammatory reactions, acute respiratory insufficiency or kidney failure
- How to deal with the causes and consequence of the various complications

In all of this, the methodology of clinical epidemiology, which is the tool that generates the notions of evidence-based medicine and evidence-based health, is fundamental. Thus, to evaluate various risk factors, observational studies are extremely useful. These may include case-control studies, which can be applied to ascertain whether age, medications, profession or attitudes might have an association with becoming ill with the disease. These studies have the great advantage of enabling assessment of several risk factors in a single study and can be conducted quickly. A very good case-control study was published recently.^2^

On the other hand, because these studies are retrospective, they have the disadvantage of depending on the patients’ memory. Moreover, they are not of much use for evaluating treatments and diagnostic tests because the proportions of cases and controls to be compared are established in an artificial manner.

For prospective evaluation of patients’ evolution and what the risk factors associated with their evolution are, prospective cohort studies are fundamental. For example, it might be asked whether COVID-19 has outcomes of greater severity in patients whose laboratory assay results are more inclined to the left or to the right. In such cases, prospective studies that have been suitably designed beforehand will supply data and evidence that are much more trustworthy than those from retrospective analyses on cases that have already occurred but were not followed up in a planned manner. Prospective studies will thus avoid data losses.

It might be asked whether patients with higher C-reactive protein levels in blood analyses are at greater risk of mortality. Prospective cohort studies frequently have the disadvantage of requiring very long and laborious follow-ups. However, this does not occur significantly with COVID-19, given that this disease only lasts for two to four weeks. Thus, within a short time, large numbers of cases within this pandemic can be evaluated prospectively.

A variety of associations of risk factors for worsening of COVID-19, obtained through retrospective studies, have now been reported.^3^^,^^4^ However, the strength of the evidence would be much more trustworthy if these data were obtained prospectively.

After hypotheses have been raised retrospectively, knowledge can be improved through prospective studies, so as to have a much more realistic vision of what is happening with each marker. Thus, therapeutic interventions aimed towards each desirable outcome and/or a set of outcomes of interest can be planned.

Therapeutic interventions cannot be securely assessed through case series without a protocol for a controlled clinical trial. Furthermore, such trials should preferably be double-blind, and these are necessarily prospective. Even though many people are greatly attracted towards cases series, these should generally serve only to generate questions that later on need to be answered using clinical trials. Hypotheses are then tested so that treatments can be put into practice based on evidence. This is very different from conduct based only on hypotheses.

Retrospective studies or studies without control groups may show that a series of patients received a benefit, or even some harm. However, these studies always leave a trail of continuing uncertainties if they are not followed by adequate prospective studies. This is so even in situations of the best of intentions, such as in cases of compassionate treatment. We end up not knowing whether the treatment was beneficial or harmful because we did not have a comparison group that was chosen randomly and impartially.

On the other hand, a clinical trial that is conducted with the competence and seriousness that this merits will, independent of the result, bring great benefits for all of humanity. In other words: if the intervention was beneficial, it can be used; but if it was ineffective, resources and sometimes lives will not be wasted, and the way forward may then be to explore new possibilities.

Among the sources of great confusion in the literature on this subject, both in academic settings and in the lay press, lack of verification of the application of basic scientific fundaments of knowledge has generated confusion, conflict and commotion that can only be resolved through good-quality evidence.

For example, since the expected average mortality rate from symptomatic cases of COVID-19 is around 5%, it can be expected therefore that out of every 100 cases, around 95 should survive purely through the action of nature. Hence, even if only placebo is administered (which could be physiological serum or whatever else), 95% of the patients will be saved and will be computed as cases of successful treatment and the other 5% will not be able to come back to complain, in the remote hypothesis that all the cases are well documented.

Thus, in the absence of an adequate control group, masking for the researcher and patients, randomization, a good study protocol and a good way of conducting the study, large quantities of useless data end up supporting spurious opinions of well-intentioned people who are unaware of the appropriate methods for obtaining trustworthy data. This also supports the opinions of ill-intentioned people whose interest lies in deceiving others and promoting themselves professionally to gain financial advantages. Good scientific products are the fruit of a continuous struggle against possible confounding factors, systematic errors and random effects that might falsify the conclusions and mislead the researcher.

Although evidence-based medicine has led to great achievements so far, both in Brazil and around the world, there is still much to be done. The current pandemic has certainly helped to improve social awareness of the role of well-prepared scientists as essential actors in the war against this extremely dangerous invisible enemy. This is a war in which the lives of hundreds of millions of people depend on the outcome, and definitively it is not for just any healthcare professional to engage in.

On the websites of clinical trial registers, more than one hundred trials on treatments for COVID-19 are currently in progress. One of the best of these, Recovery, conducted by Oxford University,^5^ is a factorial study, i.e. with several therapeutic interventions against COVID-19 in comparison with a control group. Its aim is to produce rapid answers to the research questions, which is possible because of the enormous number of cases that have occurred and the short duration of the disease.

One of these clinical trials has compared use of oral or intravenous dexamethasone in patients hospitalized with COVID-19 who require noninvasive oxygenation or mechanical ventilation. Given the high quality of this project and the adequate sample size, there is little doubt that this trial provides the first evidence of drug treatment with real benefit for treating patients with COVID-19 who require supplementary oxygenation, with significant reduction in mortality. However, this finding does not apply to milder cases.

Other studies with similar objectives are underway and, if their quality is good, their results may have an important role in reducing the remaining uncertainties, when added carefully to the current results in a conservative statistical process and through a properly done meta-analysis. In this manner, the best level of evidence for basing healthcare decisions on will be achieved through systematic reviews, and these trials will then enter history. The Cochrane Collaboration has the role of doing analyses of this nature in an impartial and live manner, i.e. continuously and explicitly, in all important fields of healthcare, and publishing them in the Cochrane Library.

Consulting good sources of evidence is essential for patients, students, doctors, other healthcare professionals, researchers, managers, journalists and lawyers. Free access to the Cochrane Library has been available in Brazil since 2001.

The Cochrane Center of Brazil has been in operation within the Federal University of São Paulo (Universidade Federal de São Paulo, UNIFESP) since 1996 (https://brazil.cochrane.org/).
